# Effects of a Dart Game Intervention in Community-Dwelling Older Adults with Suspected Mild Dementia: An Exploratory Study Using the Japanese Version of the Montreal Cognitive Assessment

**DOI:** 10.7759/cureus.90873

**Published:** 2025-08-24

**Authors:** Tadayuki Iida, Maria Akane, Mayu Nakata, Chinami Ishizuki, Ruriko Miyashita, Asami Nishiguchi, Nariaki Hitotsubashi, Takumi Sakamoto, Shuhei Kaneko, Hideki Miyaguchi

**Affiliations:** 1 Department of Physical Therapy, Faculty of Health and Welfare, Prefectural University of Hiroshima, Mihara, JPN; 2 Department of Physical Therapy, Kashiba Asahigaoka Hospital, Nara, JPN; 3 Department of Physical Therapy, Yawata Medical Center, Ishikawa, JPN; 4 Faculty of Health Sciences, Department of Rehabilitation Science, University of Kochi Health Sciences, Kochi, JPN; 5 Department of Nursing, Women’s Health Cares Nursing Master’s Program of Midwifery Course, Kobe City College of Nursing, Kobe, JPN; 6 Department of Health Care Facilities, IKI Health Care Facilities for the Elderly Requiring Long-Term Care, Kyouseikai Medical Corporation, Nagasaki, JPN; 7 Department of Head Office Operations, ONEENTERPRISE Co. Ltd., Hiroshima, JPN; 8 Department of Store Operations, NICE DARTS CLUB, Hiroshima, JPN; 9 Department of Event Promotion, DARTSLIVE Co. Ltd., Tokyo, JPN

**Keywords:** attention, dart games, delayed recall, mild cognitive impairment, moca-j, older adult

## Abstract

Background: With the aging of the population, awareness of various dementias, including Alzheimer's disease, is spreading. The causes of these dementias are being elucidated, while prevention is also becoming more important. Aerobic exercise, strength training, and flexibility exercise (physical exercise) alone are not sufficient to improve cognitive function, and dual-task activities that engage both physical and cognitive functions are effective for its prevention. Therefore, the present study investigated the effects of an intervention with dart games on cognitive function in older adults suspected of having mild cognitive impairment (MCI) who reside in the community. Factors contributing to these effects were also examined.

Methods: The intervention with dart games lasted for six months and was tested on healthy older individuals (aged ≥ 65 years) recruited from the community (Mihara City and Kumano Town in Hiroshima Prefecture and Iki City in Nagasaki Prefecture). The dart game intervention was the Count-Up and 01 Game, which took place every two weeks over a six-month period, with each session lasting 100 minutes. Data from 62 participants with or without MCI were analyzed. An intervention was conducted in which older adults suspected of having MCI who reside in the community played dart games for six months. Cognitive function was assessed before and after the intervention using the Montreal Cognitive Assessment-Japanese version (MoCA-J) to evaluate the effects of the intervention. Participants were classified based on their MoCA-J scores, and those scoring ≤25 were categorized as suspected MCI. For those classified as suspected MCI before the intervention, it was determined whether they were MCI (non-improved MCI group) or not MCI (improved MCI group) after the intervention. In both groups, MoCA-J subdomain scores (orientation (six points), language (three points), visuospatial/executive function (five points), delayed recall (five points), abstraction (two points), attention (six points), and naming (three points)) were compared. The predictive ability of MoCA-J scores for MCI improvement due to the dart game intervention was evaluated using the area under the receiver operating characteristic (ROC) curve (AUC).

Results: Among patients classified as suspected MCI before the intervention (n=27), 11 were in the non-improved MCI group and 16 in the improved MCI group after the intervention, with some subjects showing improvement in cognitive function following the intervention. In a comparison of the MoCA-J subdomain scores before and after intervention for those classified as suspected MCI before the intervention, improvements were revealed in “delayed recall (non-improved MCI group 0.0 vs. improved MCI group 2.5, median comparison)” and “attention (non-improved MCI group 0.0 vs. improved MCI group 1.0, median comparison)”. The predictive ability of the dart game intervention to improve MCI for subjects classified as MCI before the intervention was determined using an ROC curve, with an AUC of 0.841. The optimal cut-off score based on Youden’s index was 22 points.

Conclusion: The dart game intervention demonstrated potential cognitive benefits, particularly in brain regions associated with “delayed recall” and “attention.” Among older adults with MoCA-J scores of approximately 22, participating in a six-month dart game intervention may help improve MCI.

## Introduction

In 2018, the average life expectancy in Japan was 81.25 years for men and 87.32 years for women, making it one of the top-ranking countries for longevity worldwide. With the extension of life expectancy, the percentage of older adults, which was approximately 5% in 1960, significantly increased to 28.4% in 2019 (13.8% for those aged between 65 and 74 years and 14.6% for those aged 75 years and older), with the population aged 75 years and older surpassing that aged between 65 and 74 years. The number of individuals aged 75 years and older in Japan is expected to reach 22 million by 2030, an unprecedented figure globally [1]. Furthermore, Japan’s Ministry of Health, Labour, and Welfare estimated that the number of older adults with dementia was 4.62 million in 2012 and is projected to increase to approximately seven million by 2025, meaning that one in five individuals aged 65 years and older will have dementia. Similarly, the World Health Organization (WHO) predicts that due to global population aging, the number of individuals living with dementia will triple from 50 million currently to 152 million by 2050 [2]. Therefore, addressing dementia is an urgent issue not only in Japan but also worldwide.

In anticipation of 2025, when the baby boomer generation in Japan will be 75 years or older, the Ministry of Health, Labour, and Welfare formulated the “Comprehensive Strategy for Dementia Measures-Toward a Dementia-Friendly Community” (New Orange Plan) in 2015 [3]. The New Orange Plan aims to create a society where individuals with dementia may live with dignity and as independently as possible in their familiar communities. The key initiatives of the New Orange Plan include “support for individuals with dementia through collaborative efforts between medical and long-term care facilities,” “creating dementia-friendly communities,” and “research and development for dementia prevention and treatment.”

One of the key factors in delaying the onset of dementia is addressing modifiable risk factors [4]. The primary modifiable risk factors for dementia among older adults living in rural areas include social isolation due to staying at home, leading to a decline in both cognitive and physical functions [5-10].

Previous studies showed that social interaction and participation in daily activities reduced the need for long-term care by 50% and lowered the incidence of dementia by 30% [11]. Furthermore, promoting complex activities that include physical exercise has been recognized as an effective dementia prevention strategy. Studies suggest that a combined approach is more effective for cognitive function than physical exercise or cognitive training alone [12-14]. Numerous intervention studies have reported cognitive improvements in individuals with mild cognitive impairment (MCI) through multicomponent programs combining physical activity, cognitive training, and social engagement [15, 16]. Among such approaches, recreational interventions such as dart games have garnered attention due to their ability to combine motor and cognitive challenges in a socially interactive format [17]. When a person aims and throws at a target, several brain regions are involved, including the visual cortex, temporal association cortex, parietal association cortex, and prefrontal cortex, in order to recognize the target, process somatosensory input, and select the spatial position. The prefrontal cortex then makes the decision to throw, and based on this information, the motor association cortex plans the most efficient throwing movement, which is executed via motor output from the primary motor cortex through the spinal cord. During subsequent throws, the parietal association cortex and cerebellum compare the predicted outcome with actual sensory feedback to extract errors [18]. Motor learning subsequently occurs by planning how to refine the movement most effectively for future attempts [19].

Schleien et al. demonstrated that in handicapped adults, aiming at a target while performing calculations improved dart-playing skills and enhanced sensory feedback effectiveness [20]. Therefore, dart games integrate both physical activity and cognitive tasks, allowing participants to perform movement and calculation tasks simultaneously [17]. Unlike repetitive exercises with predetermined motions, darts is classified as a sport that incorporates enjoyment and game elements. Dart throwing does not rely solely on arm strength; it also requires lower limb and core stability, balance control, spatial cognition, attention, and calculation skills, making it a dual-task sport that engages both physical and cognitive functions [21]. In contrast to repetitive or monotonous training, darts is characterized by enjoyment, novelty, and interpersonal interaction, which are known to promote adherence and long-term engagement in older adults [22,23]. Furthermore, the adaptable nature of darts for individual or group formats supports both independent task execution and socially facilitated participation [21].

In this context, we propose dart games as a novel, enjoyable, and socially engaging dual-task intervention with the potential to improve cognitive function in older adults with suspected MCI. To our knowledge, few studies have directly investigated the effectiveness of dart-based programs in this population. Therefore, this study aimed to evaluate the impact of a dart game intervention on cognitive function among community-dwelling older adults with suspected MCI. The intervention was conducted at community centers and similar venues where older adults regularly gather, enabling naturalistic participation and interaction. Community-based venues offer a familiar and accessible environment that reduces psychological and logistical barriers to participation.

## Materials and methods

Participants

We enrolled healthy adults aged 65 years and older residing in Mihara City and Kumano Town in Hiroshima Prefecture, as well as in Iki City in Nagasaki Prefecture, who were not utilizing long-term care insurance services. Adults aged 65 years or older account for approximately 36% of Mihara City, 35.7% of Kumano Town in Hiroshima Prefecture, and 39.3% of Iki City in Nagasaki Prefecture. Recruitment was conducted through public relations bulletins issued by the town and city offices. The public relations bulletins stated that one of the evaluation criteria was that the participant be diagnosed with dementia or other mental disorders, such as depression, and participants applied to the researcher after reading this public relations bulletin. Exclusion criteria were as follows: (1) individuals with difficulty in verbal communication who were unable to respond to questions, and (2) individuals for whom study-related assessments were challenging to perform. Participants received a detailed explanation of the objectives and procedures of the study at an informational session, after which written informed consent was obtained from 77 individuals. Among them, three participants were excluded based on the pre-intervention assessment due to meeting the exclusion criteria, six did not participate in the post-intervention assessment, and six were excluded due to refusal or missing questionnaire responses in either the pre- or post-intervention assessments. As a result, 62 participants were included in the final analysis. This study was conducted in accordance with the principles of the Declaration of Helsinki and was approved by the Ethics Committee of Hiroshima University (approval number: No. 18MH031-01).

Study schedule

The intervention period was six months. In Mihara City, Hiroshima Prefecture, the intervention was conducted between October 2019 and March 2020, while in Kumano Town, Hiroshima Prefecture, and Iki City, Nagasaki Prefecture, it took place between March 2022 and September 2022. Comparison of pre-intervention age, height, and weight in each region is shown in Table 1. From this, no regional differences were found. Also shown is the presence or absence of suspected MCI using the Japanese version of the Montreal Cognitive Assessment (MoCA-J) before the intervention in each region (Table 2). No regional differences were found. Cognitive function was assessed both before and six months after the intervention.

**Table 1:** Comparison of age, height and weight in the three regions †: one-way ANOVA

Variables	Mihara City		Kumano Town		Iki City		
	Mean	SD	Mean	SD	Mean	SD	p-value†
Age (years)	74.7	6.6	72.0	6.6	74.7	6.3	0.424
Height (cm)	157.1	10.2	153.1	5.6	157.4	8.3	0.270
Weight (kgs)	56.4	9.5	57.2	7.0	58.2	13.5	0.842

**Table 2:** Comparison of the suspected mild cognitive impairment (MCI) and non-MCI cases in the three regions before the intervention. †: Fisher's exact test

Variable	MCI status	Mihara City	Kumano Town	Iki City	p-value†
Pre-intervention	Suspected MCI cases	16	5	6	1.000
	Non-MCI cases	20	7	8	

Dart Games

In Kumano Town (Hiroshima Prefecture), dart games were played as part of an exercise program for older adults organized by the local community gymnasium. Dart game sessions were held biweekly over a six-month period, with each session lasting 100 minutes. Participants formed teams of either four or two players. The program utilized two dartboards (Darts Live2 EX, manufactured by DARTSLIVE Co. Ltd., Tokyo, Japan). Instructors participated in each session, providing guidance on dart-throwing techniques based on an instructional plan developed in consultation with individuals holding professional dart qualifications.

In Mihara City (Hiroshima Prefecture) and Iki City (Nagasaki Prefecture), dartboards were installed in a local community center, where members of a community-based social group engaged in dart games. These members had free access to the dartboards. Every three months, during assessment periods, an individual with a professional dart qualification provided instructions on dartboard operation, throwing techniques, and game rules.

Two types of dart games were played: Count-Up and 01 Game. Count-Up is a competitive game in which players aim for the highest cumulative score. Each round consists of three dart throws, and the game continues for eight rounds, with the winner having the highest total score. 01 Game starts with a set score (e.g., 301 or 501 points), and players aim to reduce their points to exactly 0. If a player scores below zero, they cannot complete the round successfully. Since both games require calculations and precise targeting, they serve as dual-task activities that simultaneously engage cognitive and motor functions.

Measurements

Patient demographic information, including sex, age, and years of education, was collected. The MoCA-J is the Japanese version of the Montreal Cognitive Assessment (MoCA) [24], originally developed by Nasreddine et al. [25], and was translated and culturally adapted into Japanese with permission from the original author. The MoCA is a brief cognitive screening tool designed to detect mild cognitive impairment [24]. It assesses multiple cognitive domains with the following maximum subdomains scores: Orientation (six points), Language (three points), Visuospatial/executive function (five points), Delayed recall (five points), Abstraction (two points), Attention (six points), and Naming (three points), totaling 30 points. In accordance with the original scoring guidelines (the official website mocatest.org), one additional point was added to the total score for participants with 12 or fewer years of formal education to correct for educational bias. The Japanese version (MoCA-J) has been validated in community-dwelling older adults in Japan, demonstrating excellent diagnostic accuracy, with a sensitivity of 93.0% and specificity of 87.0% using a cut-off score of 25/26 [24]. These results support the use of the MoCA-J as a reliable and effective screening tool for identifying individuals with suspected MCI. Changes in each subdomain were calculated as the difference between post- and pre-intervention scores. Positive values indicate improvement, while negative values indicate a decline in the respective cognitive function.

Classification of suspected MCI and non-MCI groups

Participants were classified based on their MoCA-J scores, and those scoring ≤25 points were categorized as suspected MCI. The non-improved MCI group included participants who were classified as suspected MCI before the intervention and remained in this category after the intervention. The improved MCI group consisted of individuals initially classified as suspected MCI who, after the intervention, were no longer classified as suspected MCI. The deteriorating non-MCI group comprised participants who were initially classified as non-MCI, but fell into the suspected MCI category after the intervention. The maintained non-MCI group included individuals classified as non-MCI before the intervention who remained non-MCI post intervention.

Sample size

The sample size was determined according to the two-week difference in MoCA score using G-power statistical software (Ver. 3.1 Heinrich-Heine-Universität Düsseldorf, Düsseldorf, Germany), on the basis of Li et al.’s results [26]. The comparison of cognitive training intervention and non-intervention in the mild dementia group, with M1 = 6.39 (mean MoCA changing score of experimetal), SD 1 = 2.96, M2 =1.17 (mean MoCA changing score of control), SD 2 = 1.05, two-sided α = 0.05, and power = 95%, as well as two-sided α = 0.05 and power = 95%, the sample size per group was set as nine. In the present study, the smallest sample size in the improved MCI group was 11. The minimum sample size is therefore satisfied.

Statistical Analysis

A chi-square test was used to examine the relationship between the suspected MCI classification before and after the dart game intervention. A one-way ANOVA was conducted to compare age differences among the classification groups, followed by Bonferroni post-hoc multiple comparisons. The Mann-Whitney U test was used to compare MoCA-J subdomain scores between the non-improved and improved MCI groups. The predictive ability of pre-intervention MoCA-J scores for MCI improvement was evaluated using the area under the receiver operating characteristic (ROC) curve (AUC), and Youden’s index was used to select the optimal cut-off point where sensitivity and specificity were maximized.

## Results

Comparison of participants before and after the intervention

Table 3 shows changes in suspected MCI and non-MCI classifications before and six months after the intervention. The chi-square test revealed a correlation between the dart game intervention and suspected MCI status (p=0.015).

**Table 3:** Suspected mild cognitive impairment (MCI) cases before and after the dart game intervention

Variable	MCI status	Post intervention		
		Suspected MCI cases	Non-MCI cases	p-value
Pre-intervention	Suspected MCI cases	11	16	0.015
	Non-MCI cases	4	31	

Age comparison

A one-way ANOVA analysis revealed significant differences in age among the groups. Bonferroni post-hoc multiple comparisons indicated a significant difference between the non-improved MCI group and the maintained non-MCI group (Table 4). However, no significant age difference was observed between the non-improved MCI group and the improved MCI group.

**Table 4:** Relationship between suspected mild cognitive impairment (MCI) and age p value: A one-way analysis of variance (ANOVA); †1: the Bonferroni method; p=0.004

Suspected MCI	Number	Mean	(SD)		p-value
Non-improved MCI	11	79.2	(8.2)	^†1^	0.005
Improved MCI	16	73.7	(3.4)		
Deteriorating non-MCI	4	71.0	(3.5)		
Maintained non-MCI	31	72.2	(5.4)	^†1^	

Comparison of MoCA-J subdomains in participants with suspected MCI

Table 5 shows a comparison of MoCA-J subdomain scores before the intervention between the non-improved MCI group and the improved MCI group. A significant difference was observed in the delayed recall subdomain (p=0.011). Table 6 shows changes in MoCA-J subdomain scores before and after the intervention. Significant differences were found in delayed recall (p<0.001) and attention (p=0.027) between the two groups.

**Table 5:** Pre-intervention Montreal Cognitive Assessment-Japanese version (MoCA-J) subdomains in non-improved mild cognitive impairment (MCI) and improved MCI groups p-value: Mann-Whitney U test; test statistic: standardized test statistic; r: standardized test statistic scaled by the square root of the sample size (√n)

Variable	Non-improved MCI group (n=16)				Improved MCI group (n=11)						
MoCA-J subdomains	Median	Min	-	Max	Median	Min	-	Max	p-value	Test statistic	r
Orientation	6.0	2.0	-	6.0	6.0	5.0	-	6.0	0.109	2.583	0.497
Language	1.0	0.0	-	3.0	1.5	0.0	-	3.0	0.055	1.942	0.374
Visuospation/Executive function	4.0	1.0	-	5.0	4.0	2.0	-	5.0	0.266	1.139	0.219
Delayed recall	0.0	0.0	-	3.0	2.0	0.0	-	5.0	0.011	2.583	0.497
Abstraction	2.0	0.0	-	2.0	2.0	1.0	-	2.0	0.143	1.500	0.289
Attention	5.0	3.0	-	6.0	5.0	4.0	-	6.0	0.509	0.689	0.133
Naming	3.0	1.0	-	3.0	3.0	2.0	-	3.0	0.324	1.023	0.197

**Table 6:** Pre- and post-intervention Montreal Cognitive Assessment-Japanese version (MoCA-J) subdomains in non-improved mild cognitive impairment (MCI) and improved MCI groups p-value: Mann-Whitney U test; test statistic: standardized test statistic; r: standardized test statistic scaled by the square root of the sample size (√n)

Variables	Non-improved MCI group (n=16)				Improved MCI gruop (n=11)						
MoCA-J subdomains	Median	Min	-	Max	Median	Min	-	Max	p-value	Test statistic	r
Change in orientation	0.0	-2.0	-	2.0	0.0	-1.0	-	1.0	0.841	0.229	0.044
Change in language	0.0	-2.0	-	2.0	1.0	-1.0	-	2.0	0.367	0.927	0.178
Change in visuospation/Executive function	0.0	-1.0	-	3.0	0.5	-2.0	-	3.0	0.160	1.431	0.275
Change in delayed recall	0.0	-3.0	-	1.0	2.5	-1.0	-	5.0	<0.001	3.632	0.699
Change in abstraction	0.0	-1.0	-	0.0	0.0	0.0	-	1.0	0.204	0.328	0.063
Change in attention	0.0	-4.0	-	1.0	1.0	-1.0	-	1.0	0.027	2.234	0.430
Change in naming	0.0	-1.0	-	2.0	0.0	-1.0	-	1.0	0.799	0.291	0.056

ROC analysis of MoCA-J scores and MCI improvement

**Figure 1:**
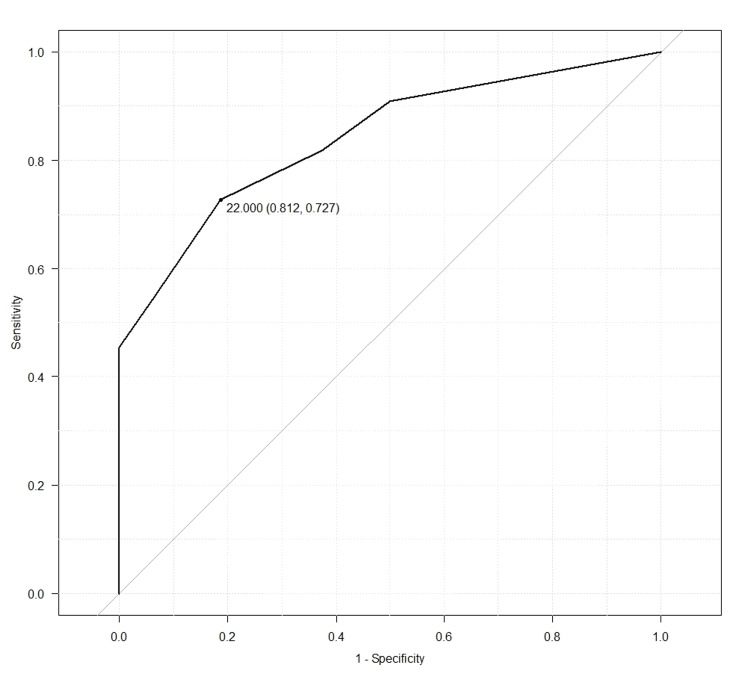
Receiver operating characteristic (ROC) for pre-intervention Montreal Cognitive Assessment-Japanese version (MoCA-J)

Figure 1 shows the results of the ROC analysis examining the relationship between MoCA-J scores and improvements following the dart game intervention. The AUC was 0.841, indicating good discriminative ability. The optimal cut-off score based on Youden’s index was 22 points.

## Discussion

In the present study, we compared cognitive function before and after the six-month intervention to examine the relationship between dart games and the presence or absence of suspected MCI. Among the 27 participants classified as suspected MCI before the intervention, 16 showed improvement. The results of the chi-square test indicated a correlation between dart game participation and cognitive function changes. The group-based nature of the dart game, which fosters social communication [11], and its characteristics as a dual-task sport involving both physical and cognitive functions [12-14, 20] likely contributed to the observed cognitive improvements.

To further analyze these effects, we focused on participants classified as suspected MCI before the intervention and divided them into the non-improved and improved groups based on their post-intervention status. A significant difference between these groups was observed in the delayed recall subdomain of MoCA-J before the intervention. Additionally, significant differences were noted in delayed recall and attention between pre- and post-intervention scores. Delayed recall is associated with frontal and parietal lobe functions, while attention involves the frontal lobe, parietal lobe, and prefrontal cortex. Throwing darts requires precise spatial recognition between the player and the target, similar to the process of visually aiming at a target. When a target is identified, visual information is transmitted from the occipital lobe to the superior parietal lobule in the parietal association cortex, where it is integrated with somatosensory information to identify the target’s spatial position and guide body coordination. This information is then relayed to the dorsal premotor area, where the most efficient throwing motion is planned. Visual information is simultaneously sent to the inferior parietal lobule and ventral premotor area, where the optimal grip pattern for throwing is selected and planned. This motor plan is then transmitted as output from the primary motor cortex to the spinal cord. In other words, recognizing the target and identifying its spatial position requires frontal and parietal lobe functions, which may explain why a significant difference was observed in delayed recall between the non-improved and improved groups before the intervention. Additionally, after a throw, the parietal association cortex and cerebellum compare the predicted motor outcome with actual sensory feedback, extracting any errors [18]. Dart motion feedforward under these conditions is converted into predictive information about the motor outcome, and the parietal association cortex and cerebellum compare the predicted outcome with actual sensory feedback to extract errors. The result is motor-based learning to efficiently perform the motor task of throwing darts. As aging progresses, motor speed declines, and aiming at distant targets requires not only arm movement, but also forward weight shifting. Older adults also experience a decline in hand-eye coordination and motor control, leading to greater variability in movement. Repeated exposure to dart-throwing tasks helps reinforce motor learning, allowing participants to improve efficiency in throwing over time [20]. Repeated exposure to dart games and motor learning leads to subsequent dart-throwing tasks. The frontal lobe is responsible for attention, thought processing, and emotional control, and is involved in the ability to organize, process, and execute. Therefore, the improvements observed in delayed recall and attention in the improved MCI group may be attributed to the continued practice and repetition of these processes.

The ROC analysis evaluating the predictive ability of pre-intervention MoCA-J scores for MCI improvement yielded an AUC of 0.841. Based on this analysis, a MoCA-J cut-off score of 22 appeared to be an appropriate threshold. These results suggest that the dart game intervention is particularly effective for older adults with MoCA-J scores of approximately 22. A previous study showed that in logistic regression analyses conducted 1 month after the intervention, age was identified as a significant risk factor for cognitive decline in individuals with MCI [27]. In Japan, the prevalence of dementia is estimated to be 10% to 15% in individuals in their early 80s, 15% to 25% in those in their late 80s, and >30% in those aged 90 years and older [28]. Therefore, aging remains the greatest risk factor for dementia. The present results are consistent with these findings, showing that the likelihood of cognitive improvement decreases with increasing age at MCI onset. Therefore, the primary prevention of MCI may be more important than secondary or tertiary prevention. The present results suggest that introducing dart games as an intervention for healthy older adults at the early stages of MCI helps prevent cognitive decline.

Study limitations and future research

One limitation of this study is that the participants were self-selected volunteers, likely reflecting a group with higher health awareness. In addition, as the diagnosis of dementia or other mental disorders, such as depression, was based on self-report, we cannot rule out the possibility that some individuals with such conditions were included in the study population. Additionally, the MCI classification criteria vary across studies [29-31]. Petersen, who originally established the concept of MCI, reported unresolved issues regarding the choice of cognitive assessment tools and the criteria for distinguishing normal cognition from MCI [32]. Despite these challenges, we used MoCA-J because it has been shown to have higher sensitivity and specificity for detecting MCI than the Mini-Mental State Examination [24, 33, 34]. Moreover, although MoCA-J was used for both pre- and post-intervention assessments of cognitive function, the use of the same cognitive measure before and after the intervention raises the possibility that practice effects may have influenced the results. By categorizing participants into the MCI and non-MCI groups and applying a standardized physical intervention, we were able to obtain valuable insights into both protective and risk factors for MCI development.

## Conclusions

The dart game intervention demonstrated potential cognitive benefits, particularly in brain regions associated with movement-based cognitive functions. This intervention engages postural control, balance, spatial cognition, and weight shifting, making it an effective dual-task training activity. Among older adults with MoCA-J scores of approximately 22, participating in a six-month dart game intervention may help improve MCI.
